# Use of Mobile Health Apps and Wearable Technology to Assess Changes and Predict Pain During Treatment of Acute Pain in Sickle Cell Disease: Feasibility Study

**DOI:** 10.2196/13671

**Published:** 2019-12-02

**Authors:** Amanda Johnson, Fan Yang, Siddharth Gollarahalli, Tanvi Banerjee, Daniel Abrams, Jude Jonassaint, Charles Jonassaint, Nirmish Shah

**Affiliations:** 1 Department of Pediatrics Duke University Durham, NC United States; 2 Department of Computer Science & Engineering Wright State University Dayton, OH United States; 3 North Carolina State University Raleigh, NC United States; 4 Engineering Sciences and Applied Mathematics Northwestern University Chicago, IL United States; 5 Social Work and Clinical and Translational Science Department of Medicine University of Pittsburgh Pittsburgh, PA United States; 6 Division of Hematology Department of Medicine Duke University Durham, NC United States

**Keywords:** pain, sickle cell disease, SCD, machine learning

## Abstract

**Background:**

Sickle cell disease (SCD) is an inherited red blood cell disorder affecting millions worldwide, and it results in many potential medical complications throughout the life course. The hallmark of SCD is pain. Many patients experience daily chronic pain as well as intermittent, unpredictable acute vaso-occlusive painful episodes called pain crises. These pain crises often require acute medical care through the day hospital or emergency department. Following presentation, a number of these patients are subsequently admitted with continued efforts of treatment focused on palliative pain control and hydration for management. Mitigating pain crises is challenging for both the patients and their providers, given the perceived unpredictability and subjective nature of pain.

**Objective:**

The objective of this study was to show the feasibility of using objective, physiologic measurements obtained from a wearable device during an acute pain crisis to predict patient-reported pain scores (in an app and to nursing staff) using machine learning techniques.

**Methods:**

For this feasibility study, we enrolled 27 adult patients presenting to the day hospital with acute pain. At the beginning of pain treatment, each participant was given a wearable device (Microsoft Band 2) that collected physiologic measurements. Pain scores from our mobile app, *Technology Resources to Understand Pain Assessment in Patients with Pain,* and those obtained by nursing staff were both used with wearable signals to complete time stamp matching and feature extraction and selection. Following this, we constructed regression and classification machine learning algorithms to build between-subject pain prediction models.

**Results:**

Patients were monitored for an average of 3.79 (SD 2.23) hours, with an average of 5826 (SD 2667) objective data values per patient. As expected, we found that pain scores and heart rate decreased for most patients during the course of their stay. Using the wearable sensor data and pain scores, we were able to create a regression model to predict subjective pain scores with a root mean square error of 1.430 and correlation between observations and predictions of 0.706. Furthermore, we verified the hypothesis that the regression model outperformed the classification model by comparing the performances of the support vector machines (SVM) and the SVM for regression.

**Conclusions:**

The Microsoft Band 2 allowed easy collection of objective, physiologic markers during an acute pain crisis in adults with SCD. Features can be extracted from these data signals and matched with pain scores. Machine learning models can then use these features to feasibly predict patient pain scores.

## Introduction

### Background

Sickle cell disease (SCD) is a hematologic disorder that can cause a multitude of complications throughout a patient’s life, with pain being the most common and a significant cause of morbidity. The pain experienced by SCD patients is often chronic with acute vaso-occlusive crises that are unpredictable and lead to frequent visits to the emergency department (ED) and day hospital for management [1]. Of these patients, 1 in 4 will be admitted and can result in unplanned hospitalizations with missed days from work and school, significantly impairing a patient’s quality of life [2]. Acute pain management is palliative, with hydration and pain control via narcotic and nonsteroidal anti-inflammatory drugs (NSAIDs). With pain being inherently subjective, both medical providers and patients express difficulty in determining ideal treatment and management strategies for pain.

In the last several years, there has been an increasing focus on developing and implementing individualized pain plans [3]. However, in addition to the slow adoption of these individualized plans, difficulty also lies in understanding the patient’s degree of pain and response to pain management. With at least 1 in 4 patients with SCD seen in the ED being admitted to the hospital, it is critical to determine accurately which patients require additional pain management and which patients can be discharged.

More recently, technology has been leveraged to use mobile apps for recording symptoms in real time and wearable devices to provide more frequent physiologic measurements. The field of mobile health (mHealth) has continued to grow and has been used in a variety of different clinical settings. Many studies have attempted to help patients and providers connect using mobile technology to better understand and treat a multitude of symptoms, including pain [4-6]. Many of the initial mHealth systems and apps are smartphone-based and allow patients to self-report symptoms and activity in addition to recording objective data [7-9].

We previously reported the usefulness and validity of our mHealth app for patients with SCD [7-9]. The app has undergone multiple upgrades in the user interface based on feedback, as we continue to foster patient engagement. We have included additional health and mood questions, and the app was recently expanded to specific patient populations including bone marrow transplant patients [10]. In this study, we used Technology Resources to Understand Pain (TRU-Pain) app, which allows patients to record pain and other symptoms throughout their treatment, as described above [7]. In addition, TRU-Pain now allows the integration of wearable devices such as the Microsoft Band 2 to passively obtain physiologic data such as heart rate (HR), accelerometer activity, and galvanic skin response (GSR) using the AppleCare Kit platform.

In the face of the continued opioid crisis, the search for more objective measures of pain continues to rapidly evolve in medicine, and studies looking at a variety of objective measures to predict pain have been published in recent years. Among these studies, the types of objective data utilized to predict pain vary in invasiveness (vital signs vs neuroimaging) but show promise for utilizing such data to predict pain. Bendall et al [11] examined prehospital vital signs to predict pain severity using ordinal logistic regression and found that elevated respiratory rate, HR, and systolic blood pressure (specifically in older adults) were associated with more severe pain. A more invasive study by Lee et al used multimodal neuroimaging and HR variability with machine learning techniques to predict clinical pain in patients with chronic low back pain [12].

Owing to the growing volume of clinical data and the requirement of high accuracy predictive models, machine learning techniques have been increasingly utilized in medicine. They have been applied to multiple health care domains, from analgesic response prediction to postoperative pain estimation [13-15]. Machine learning techniques have also previously been utilized effectively in SCD studies [16,17]. Our previous study has also shown promising results in pain assessment [18]. Using nurse-obtained vital signs for patients with SCD admitted for pain crisis, our best model predicted pain with an accuracy of 0.429 on an 11-point rating scale (0 to 10) and 0.681 on a 4-point rating scale (none, mild, moderate, and severe) [18]. In these studies, machine learning can be described as a computational method to build efficient and accurate prediction models using known past information [19].

### Objectives

We now aim to use physiologic data obtained from a wearable device matched with mobile app and nurse-obtained pain scores to predict pain scores at between-subject level using machine learning techniques. The combination of mobile apps and wearable sensors has been used in several studies to provide novel solutions to different health problems [20-22]. To date, there has been a paucity of research in SCD focused on pain prediction, despite the critical need. The ability to objectively and accurately predict pain severity and onset could result in more prompt and effective treatment of pain crises, leading to improved outcomes, as well as encouraging more diligent use of medications [23,24]. Using our past experience, our hypothesis for this study was as follows: For SCD patients presenting in acute pain, can we feasibly obtain objective data from a wearable device and then utilize machine learning techniques to accurately predict pain scores?

## Methods

### Recruitment and Data Collection

Following Duke Institutional Review Board approval, patients presenting for acute pain crisis to the day hospital were approached and asked to participate in the study. A convenience sample of eligible patients who were willing to participate was consented. A small number of patients approached declined to participate, but this specific number was not recorded, and no patients withdrew from the study after consent. Of the 27 patients consented, 20 were included in this study because of insufficient data from the wearable device in 7 patients. Patients were consented Monday through Friday based on the availability of study team members. Study duration was variable based on patient’s length of stay in the day hospital. The study included a one-time visit only. Patients might have had other chronic medical conditions but were not excluded based on these conditions, and subgroup analysis was not undertaken.

Following consent, a Microsoft Band 2 wearable was placed on the patient’s wrist. The Microsoft Band 2 is a commercially available smart band that is compatible with many smartphones; it has multiple objective sensors including HR monitor, a 3-axis accelerometer and gyrometer, a GSR sensor, and a skin temperature sensor. The physiologic and activity measures utilized in the study are shown in Textbox 1. Overall, we adopted 8 wearable sensor signals to estimate pain scores (HR, R-R interval [RR; time between peak of QRS complex of electrocardiogram to subsequent QRS electrocardiogram peak], GSR, skin temperature, accelerometer [Z axis], angular velocity [Y axis], angular velocity [Z axis], and steps). These 8 signals were chosen partially based on signals readily available on the Microsoft Band 2 as well as previously postulated physiologic correlations with pain. Patients in more pain typically experience higher HR and will move less frequently in the setting of pain [25,26]. Furthermore, greater RR variability has been correlated with better pain treatment outcomes [27]. However, these objective measures have not been well established on their own to correlate with pain. Previous study by our group has supported the use of temperature, systolic blood pressure, diastolic blood pressure, oxygen saturation, and respiratory rate as statistically significant predictors in pain for SCD patients [18].

Patients were also provided with an iPad with the TRU-Pain app to record pain scores and other symptoms in conjunction with nurse-reported pain scores using a visual analog scale from 0 (none) to 10 (worst). Each patient was instructed on the use of the TRU-Pain app. The TRU-Pain app allowed patients to use a slider bar to rate their pain on the visual analog scale from 0 to 10. The app also allowed patients to note other symptoms and rate general health and mood (scale of 0 to 10). The TRU-Pain app implemented these general health and mood measures and a platform upgrade to AppleCare Kit, replacing our previous app, Sickle Cell Disease Mobile Applications to Record Symptoms via Technology. Nursing pain scores were assumed to be entered at the time they were obtained.

Both objective data from the Microsoft Band 2 and the TRU-Pain app were uploaded to a Health Insurance Portability and Accountability Act–compliant Citrix ShareFile cloud-based server. Patients were continuously monitored while in the day hospital, and at the time of discharge, the devices were returned. If patients were admitted, data before transfer were included even if the devices traveled with the patient during admission. Patients were not provided specific questions regarding acceptability and feasibility of participation, but the feasibility of the study was determined by the accuracy of machine learning algorithms in predicting pain scores.

**Physiologic and activity measures from Microsoft Band 2 (values for acceleration in X and Y directions equal that of Z direction—only acceleration in Z direction is included in this study; angular velocity in X direction was not correctly captured and was excluded from the dataset).**

- Heart rate
- R-R interval
- Galvanic skin response
- Skin temperature
- Acceleration in X direction
- Acceleration in Y direction
- Acceleration in Z direction
- Angular velocity in X direction
- Angular velocity in Y direction
- Angular velocity in Z direction
- Steps

### Data Preprocessing

To apply machine learning analysis on the collected wearable sensor data (physiologic and activity signals from the Microsoft Band 2 in Textbox 1), 3 data preprocessing steps need to be performed: time stamp matching, feature extraction, and feature selection. In time stamp matching, pain scores had to be matched with the wearable sensor data using the time stamp as close to the exact time of data collection as possible. However, the wearable sensor data samples were collected typically per second, and the pain scores were collected at varying times throughout the stay, with time stamp formatted in hours and minutes only. To complete this best possible match, each pain score was matched with the 1-min long wearable sensor data segment that was tracked at the same hour and minute. By assuming that pain scores usually do not change rapidly within a short period, we also matched the app pain scores without exact time matching to the wearable sensor data when the time stamp difference was less than 10 min.

We obtained 40 matched records containing a 1-min long wearable sensor data segment and a pain score from the mobile app that logged at the same (or approximately the same) period. However, a sample size of 40 was not sufficient for the intended data analysis. To further increase the sample size, we included nurse-documented pain scores in our dataset. Our group made the assumption that nurse-documented scores were similar to patient-reported scores in the app. Nurse-documented pain scores were matched with wearable sensor data using the within 10-min time stamps methodology as described above. By including nurse-documented pain scores, our final dataset contained 107 data samples (40 mobile app and 67 nursing notes).

After time stamp matching, each pain score was mapped to a 1-min long wearable sensor data segment that included 8 signals as mentioned in Textbox 1 above. As the sensor signal was recorded typically every second, a 1-min long segment having 8 signals contained 480 (8×60) data points. It is difficult to process raw sensor signals directly in any analytical task. Therefore, we transformed raw sensor signals to a more suitable data representation format by feature extraction. First, a moving average filter was applied to raw sensor signals to remove noise. The moving average filter is the most common filter in digital signal processing to reduce random noise [28]. Then, 8 statistical features (as described in Table 1) were extracted for each of the 8 signals. These extracted features represented the properties of the original raw signals while reducing the volume of data.

The feature extraction yielded up to total 64 (8×8) features. Given the relatively small sample size (107), a feature selection method was applied (wrapper method) to remove irrelevant or redundant features and to further reduce the number of features [29]. The wrapper method has been reported to be able to improve the predictor performance when compared with variable ranking methods [29]. The basic idea of the wrapper method is selecting the subset of features that yields the best possible performance of a given learning algorithm. A total of 2 types of search strategies are widely adopted in the wrapper method: forward selection and backward elimination. In forward selection, one starts with an empty set and features are progressively added into the subset, whereas in backward elimination, one starts with the full feature set and progressively eliminates the feature with worst performance [30].

Table 2 shows the reduced feature set using the wrapper method with forward selection. A total of 10 features were selected from 5 signals. The table also illustrates the reduced feature set with backward elimination, which contains total 14 features from 7 signals. In both feature selection approaches, no features of acceleration in Z direction (AccZ) were selected, which might be because the information contained in AccZ was already covered by Steps.

**Table 1:** List of features extracted from wearable signals.

Feature	Description
Mean	Average value of the signal
Standard deviation	Amount of variation of the signal
Mean of derivative	Average rate of change of the signal
RMS^a^	Square root of the mean of the squares of a set of values
Peak to peak	Difference between the maximum and minimum peak
Peak to RMS	The ratio of the largest absolute value to the RMS value
Number of peaks	Number of local maximums (peaks)
Power	Sum of the absolute squares of time-domain samples divided by the length

^a^RMS: root mean square.

**Table 2:** Signals and reduced feature sets.

Signal	Feature	
	Forward selection	Backward elimination
Heart rate	Mean of derivative Number of peaks	Power
R-R interval	Number of peaks	Standard deviation Peak to RMS^a^
Galvanic skin response	Mean Peak to RMS	Mean Peak to peak
Steps	Mean RMS Peak to peak	Number of peaks Power
Skin temperature	Peak to RMS	Power Mean of derivative Number of peaks
Angular velocity in Y direction	—^b^	RMS Number of peaks
Angular velocity in Z direction	—	Peak to RMS Number of peaks

^a^RMS: root mean square.

^b^Not applicable

### Machine Learning Techniques

The prediction of numeric pain score, the main study outcome, can be treated as either a regression problem or a classification problem. As the pain scores from app data are float numbers, it is more reasonable to build a regression model to provide continuous estimation of the target variable. More importantly, there is only 1 target variable (pain score) in the regression model. In contrast, there will be 11 classes if pain is treated as a classification problem, as there are 11 distinct possible pain scores (0 to 10). The number of classes can be reduced by employing a sparse rating scale. Using a widely adopted more sparse 4-point rating scale, the 11-point pain scores can be categorized as none (0), mild (1-3), moderate (4-6), and severe (7-10) [31]. However, because of our small sample size, we hold the hypothesis that the regression model is more appropriate than the classification model in this study. We adopted 4 widely used regression algorithms in our analyses: Ridge regression (Ridge), Lasso regression (Lasso), Gaussian process for regression (GPR), and support vector machines for regression (SVR). In addition, we applied support vector machines (SVM) to predict the pain scores using the 4-point rating scale and compared the results with SVR.

For linear models, we utilized Ridge and Lasso [32,33]. Linear models are easy to fit and interpret, but they cannot model the nonlinear relationships between explanatory variables and the outcome variable. The other 2 algorithms are nonlinear models. A Gaussian process (GP) is a collection of random variables such that any finite subset of them has a joint multivariate Gaussian distribution. A GP can be fully specified by a mean function and a positive definite covariance function (or kernel). GPR is one of the Bayesian learning methods in which a previous distribution over the mapping function between inputs and outputs is conditioned on observations (training process). Then, the posterior distribution can be used to make predictions [34]. GPR provides a powerful way to quantify the uncertainty in model estimations to make more robust predictions on new test data. Finally, SVM are usually applied to classification problems. In classification, the SVM model maps the input samples into the feature space, then creates a decision surface among classes with the largest distance to any data point. However, it can also be applied to regression problems where we seek to find a continuous function that maps input variables to output variables, called SVR. For SVR, the goal is to find a function that deviates from the training output by a value no greater than a certain distance for each training point, and at the same time, is as flat as possible [35]. The nonlinearity of the algorithm can be obtained by utilizing kernel modulations.

## Results

### Overview

A total of 20 adult patients (of 27 consented) had complete data. Median age was 28 years, with a range of 20 years to 66 years (Table 3). A total of 11 (11/20, 55%) patients were female, whereas 9 (9/20, 45%) were male. Moreover, 10 patients (10/20, 50%) had type SS SCD, 8 (8/20, 40%) had type SC, and 2 (2/20, 10%) had S beta thalassemia. The average length of stay in the day hospital was 3.79 (SD 2.23) hours. In addition, 2 patients were subsequently admitted to the hospital. Nursing pain scores decreased in 16 out of 20 patients (80%). Patients had an average decrease in visual analog pain score of 2.75 (SD 2.34). A total of 11 patients had multiple pain scores through the TRU-Pain app, and 91% (10/11) of the patients had a decrease in pain score, with an average decrease in pain score of 2.69 (SD 2.53).

Patients presenting to the day hospital often receive intravenous fluids, antiemetics, NSAIDs, and opioids. The opioid doses received during their day hospital stay are shown in Table 3. The last 3 columns are the number of visits each patient had to the ED and day hospital as well as admissions over the past calendar year.

**Table 3:** Patient demographics.

Patient	Age (years)	Sex	Sickle cell disease type	Insurance	Medications	Emergency department visits in prior year	Day hospital visits in prior year	Inpatient stays in prior year
1	21	F^a^	SC^b^	Public^c^	Dilaudid 6 mg; Oxycodone 5 mg	11	1	1
2	25	F	SS^d^	Public	Dilaudid 8 mg	3	8	3
3	24	F	SC	Private	Dilaudid 8 mg	1	4	3
4	40	M^e^	SS	Public	Dilaudid 16 mg; Oxycodone 5 mg	0	4	0
5	48	M	SB+^f^	Public	Dilaudid 9 mg	3	2	2
6	39	M	SS alpha^g^	Public	Dilaudid 12 mg	1	3	0
7	37	F	SC	Public	Dilaudid 9 mg	1	3	1
8	38	F	SC	Public	Dilaudid 8 mg	1	10	2
9^g^	21	M	SS	Public	Dilaudid 4 mg; Dilaudid PCA^h^	14	19	14
10	28	F	SS	Public	Dilaudid 16 mg; Oxycodone 20 mg	5	8	16
11	36	M	SS	Public	Dilaudid 6 mg	23	1	17
12	66	M	SS	Public	Dilaudid 8 mg; Morphine 4 mg	0	0	0
13	44	M	SC	Public	Dilaudid 11 mg	10	12	6
14	28	F	SB0^i^	Public	Dilaudid 8 mg	19	7	12
15	20	F	SC	Public	Dilaudid 9 mg	18	6	10
16	26	F	SS	Public	Dilaudid 13 mg	12	30	4
17	38	F	SS	Public	Dilaudid 16 mg	0	22	2
18	22	M	SC	Private	Dilaudid 8 mg	51	8	3
19	28	M	SC	Public	Dilaudid 8 mg; Oxycodone 10 mg	7	4	8
20	21	F	SS	Public	Dilaudid 5 mg; Oxycodone 10 mg	0	10	7

^a^F: female.

^b^SC: type SC (hemoglobin S and hemoglobin C).

^c^Public: at least some portion of insurance is Medicare or Medicaid.

^d^SS: type SS (hemoglobin S and hemoglobin S).

^e^M: male.

^f^SB+: type S beta thalassemia plus (hemoglobin S and beta thalassemia plus).

^g^SS alpha: type SS with alpha thalassemia (hemoglobin S and hemoglobin S with alpha thalassemia).

^h^PCA: patient-controlled analgesia.

^i^SB0: type S beta thalassemia zero (hemoglobin S and beta thalassemia zero).

### Regression Results

A total of 4 regression algorithms were implemented on 2 reduced feature sets. Results were validated using 10-fold cross-validation. Moreover, 2 evaluation metrics were applied to evaluate the performance of algorithms—the root mean square error (RMSE) and Pearson correlation [34]. RMSE is the square root of the average of squared differences between predictions and actual observations. It is measured on the same scale and has the same units as the pain score. Another metric is the Pearson correlation between predicted values and the actual values, which has a value between +1 and −1, where 0 means no linear correlation and +1 or −1 means total linear correlation. The higher the correlation value, the better the performance of the regression model. Table 4 summarizes the performance of the 4 algorithms on the 2 reduced feature sets.

For the dataset in our study, the standard deviation of 107 pain scores is 2.013, which can be interpreted as the RMSE of using the mean value as the predicted pain values. All the regression models obtained RMSE lower than the mean-only model. With 10 features in the forward selection feature set, the SVR had the best performance as the RMSE of 1.721 and the correlation of 0.522, followed by the GPR obtaining the RMSE of 1.764 and the correlation of 0.475. These results demonstrate the feasibility of using objective wearable sensor measurements to estimate subjective pain scores. With 14 features in the backward elimination feature set, the performance of GPR and SVR is further improved. The SVR model is slightly superior to the GPR model, with an RMSE of 1.430 and correlation of 0.706, respectively, which are also the best performance results obtained using regression methods. These data show that there was a strong association between the subjective pain scores (via app or nurse-obtained) and the predicted pain scores derived from wearable sensor signals.

**Figure 1:**
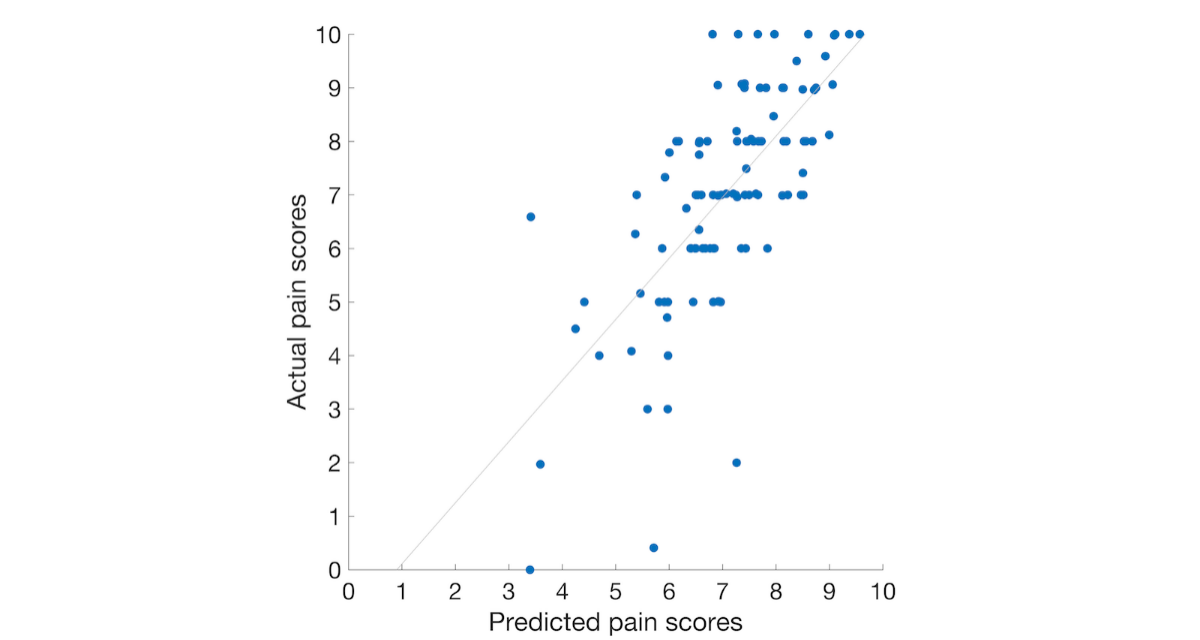
Scatter plot of the predicted and actual pain scores using the support vector machines for regression model.

The result of the SVR model with the best performance can be visualized in Figure 1. It is a scatter plot of the actual pain scores and predicted pain scores using the SVR model with the least squares regression line. The slope value of the least squares regression line is the same as the correlation of 0.706 in Table 4 and demonstrates a strong correlation of values between the actual pain scores and the predicted pain scores.

**Figure 2:**
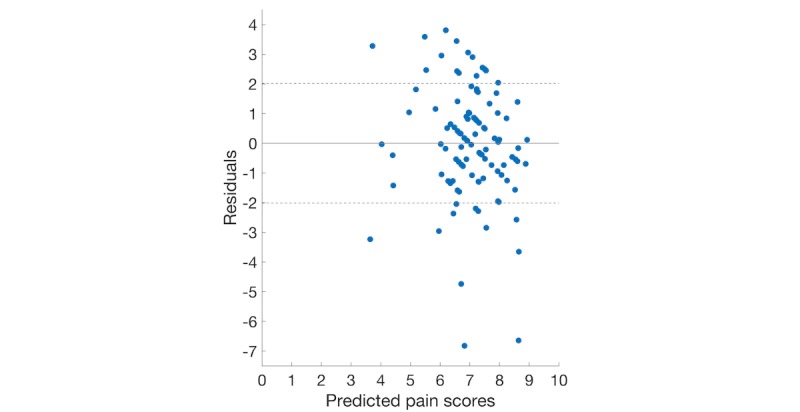
Plot of the residuals versus predicted pain scores using the backward elimination feature set.

**Figure 3:**
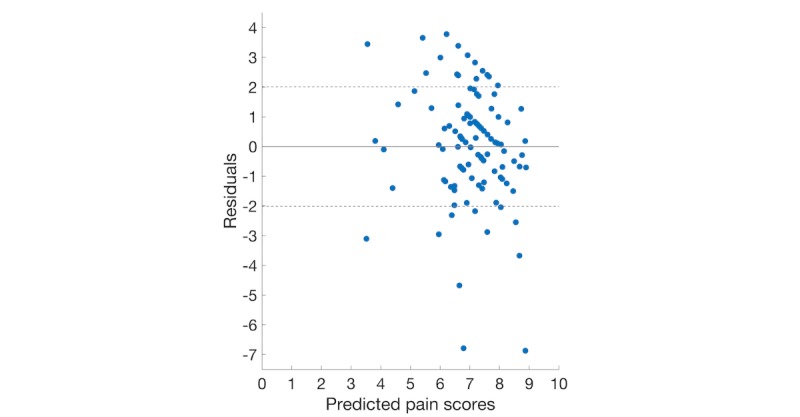
Plot of the residuals versus predicted pain scores using the backward elimination feature set (lasso).

**Figure 4:**
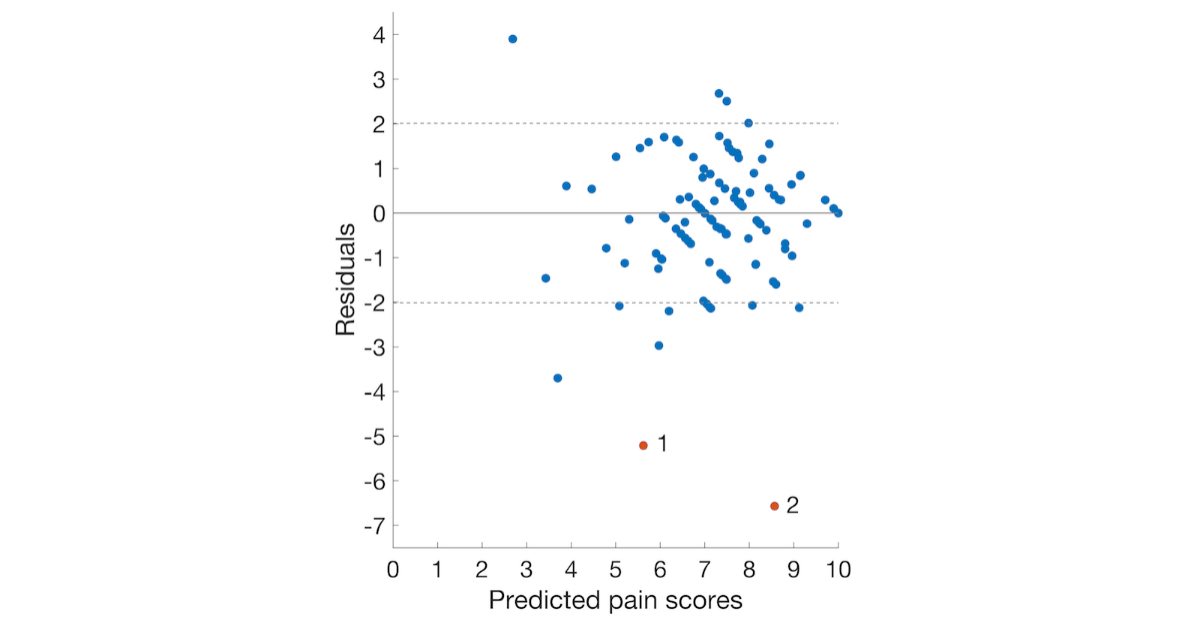
Plot of the residuals versus predicted pain scores using the backward elimination feature set (gaussian process for regression).

**Figure 5:**
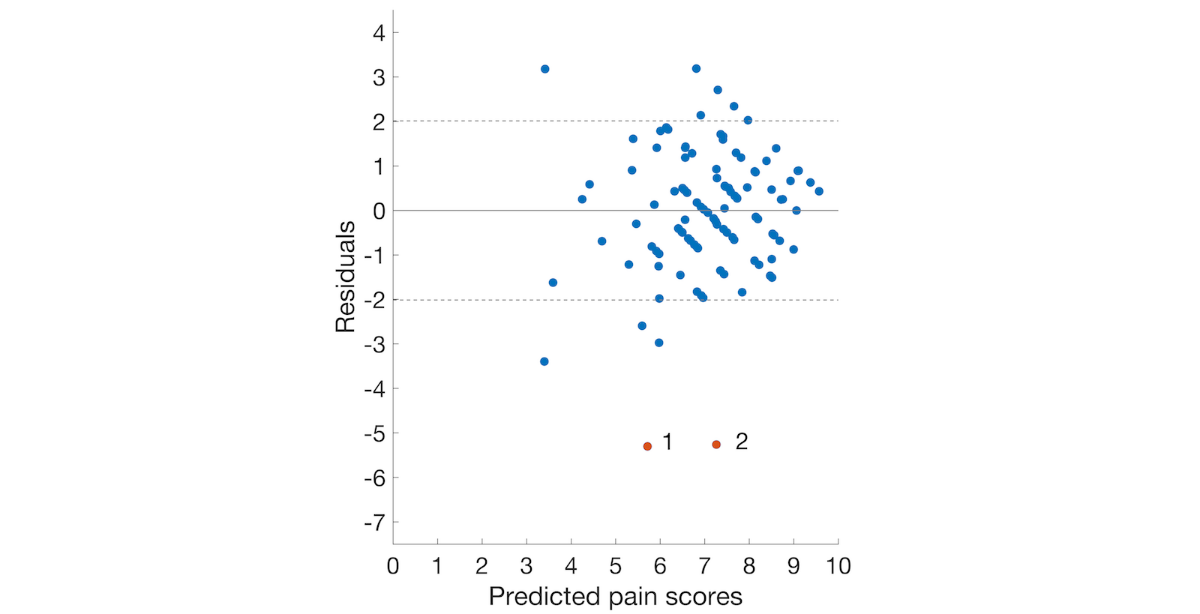
Plot of the residuals versus predicted pain scores using the backward elimination feature set (support vector machines for regression).

To better analyze the results of these regression methods, the residual plots of 4 regression models using the backward elimination feature set are illustrated in Figures 2-5. The dashed lines show the positive and negative standard deviation (2.013) of pain scores. The performances of Ridge and Lasso are nearly the same, which can be seen from Figures 2 and 3.

**Table 4:** Algorithm performances on 2 reduced feature sets using 4 regression methods.

Regression algorithm	Forward selection feature set		Backward elimination feature set	
	RMSE^a^	Correlation	RMSE	Correlation
Ridge	1.853	0.381	1.844	0.370
Lasso	1.871	0.358	1.891	0.370
Gaussian process for regression	1.764	0.475	1.473	0.683
Support vector machines for regression	1.721	0.522	1.430^b^	0.706^b^

^a^RMSE: root mean square error.

^b^Best performed model as described in the text.

In either Figure 2 or 3, there is a roughly inverted U pattern, suggesting a nonlinear relationship between predictor variables and pain scores. Thus, performances of linear models were notably lower than the other 2 nonlinear models. The distributions of residuals in Figures 4 and 5 are similar, which explains the comparable performance of the GPR model and the SVR model. The SVR model slightly surpassed the GPR model by having lower extreme residuals. Specifically, there are 2 outliers in both Figures 4 and 5, marked as points 1 and 2 (with actual pain scores of 0.41 and 2, respectively). The reason for the poor performance of these 2 points is the lack of training samples with lower pain values. It suggests that we can further improve our model performances by training the model with more samples having mild and moderate pain scores or having a larger dataset. Although a larger dataset is possible to obtain in future studies, an uneven distribution of pain scores will likely persist when acute pain crises are analyzed, as SCD patients will typically not present to medical care with lower pain scores and will manage minor crises at home [36].

### Classification Results

To apply classification to the original dataset, pain scores ranging from 0 to 10 were categorized into 4 classes as mentioned above: none (0), mild (1-3), moderate (4-6), and severe (7-10). The number of samples for the 4 pain levels are 2, 4, 34, and 67, respectively and indicates a high-class imbalance among the 4 classes. As patients visit the hospital because of pain management issues, the skewing to higher level pain scores makes clinical sense.

The SVM classifiers were applied on the categorized input dataset and evaluated for accuracy. F1 scores as well as a weighted F1 score were also evaluated. Accuracy is the ratio of correctly predicted pain scores over total number of pain scores. F1 score is the harmonic mean of precision and recall for each pain score, where precision is the ratio of the number of correctly identified entities with this pain score over the total number of this particular pain score predicted by the model. Recall is the ratio of the number of correctly identified entities with this pain score over the total number entities with this pain score in the dataset [35]. The weighted average F1 scores is the average of F1 score among all pain scores weighted by the number of instances of each pain score, and it is a better choice for evaluating datasets with multiple classes [37].

The classification result of the SVM model was compared with that of the best performance model, which was the SVR model applied on the backward elimination feature set as described above. In the experiment, both SVM and SVR were implemented on the backward elimination feature set. For a fair comparison, the same kernel was used in SVM and SVR. In addition, the continuous predicted pain scores of the SVR model were categorized into 4 classes. In this way, the accuracy, F1 scores, and weighted F1 score were calculated for the SVR model. Table 5 shows the performance comparison between the SVR model and the SVM model. Overall, the SVR model outperformed the SVM model in each evaluation metric.

From Table 5, we can see that the performance of both the SVM and SVR models were affected by the class-imbalance problem, as the F1 scores for no pain and mild pain were much lower than that for the higher pain scores. However, the SVR model can better overcome this issue by treating the outcome as a single continuous variable, as opposed to treating the prediction as a multiclass classifier. The SVR model obtained an F1 score of 0.286 for mild pain even when there were only 4 training samples with mild pain scores in the dataset. In addition, by assuming pain as a continuous variable, there are ordinal relationships between pain levels in SVR. For example, a pain score 5 is greater than pain score 4 in this model. On the contrary, the ordinal relationship is not considered in the SVM model. Treating pain as an ordinal variable is a more reasonable assumption, and it may be another reason why the regression models outperform the classification model. In summary, our results verified the hypothesis that the regression model (SVR) would obtain a higher performance than the classification model (SVM) with a small sample size and when there was a class-imbalance problem in the dataset.

**Table 5:** Prediction performances on the 4-level pain scale using support vector machines for regression and support vector machines.

Algorithm	Accuracy	F1 score of no pain	F1 score of mild pain	F1 score of moderate pain	F1 score of severe pain	Weighted F1 score
Support vector machines	0.682	0	0	0.537	0.786	0.663
Support vector machines for regression	0.729^a^	0	0.286	0.675	0.803	0.728^a^

^a^Best performed model as described in the text.

## Discussion

### Principal Findings

This study demonstrates the feasibility of using physiologic data collected on a wearable device and applying these data using machine learning techniques to accurately predict subjective pain scores. The best accuracy was found using the machine learning technique SVR, with an accuracy of 0.729 prediction of pain on a 4-point scale. In addition, for patients treated in the day hospital for pain, we found expected improvement in pain and physiologic measures such as HR from the beginning to the end of their stay.

Our predictive results are encouraging and provide insight into potential techniques to predict pain and the understanding of individual physiologic response to pain and treatment. A few investigators have recently begun to evaluate the potential use of physiologic data to develop digital phenotypes for pain and, subsequently, an individualized pain prediction model. As discussed previously, objective and physiologic data of varying invasiveness have been utilized in medicine to better understand disease processes and symptoms, including SCD. Coates et al have extensively published on objective data in SCD, including spin-tagged magnetic resonance imaging to assess cerebral oxygen extraction and metabolic rate, biventricular dimensions and function to assess cardiac iron load, and the use of a graphical Lasso model to evaluate functional brain connectivity in SCD [38-40]. This group has also published analysis of laboratory measurements of carbon monoxide and heme oxygenase for acute pain crisis prediction [41]. Other groups have studied red blood cell mechanical sensitivity and biomarker signatures of SCD severity [42,43]. The use of machine learning in a variety of areas of medicine including outcome prediction for chemoradiotherapy, breast cancer survival prediction, and early prediction of asthma exacerbations have recently been published [44-46]. However, to date, the combined use of objective and physiologic data with machine learning techniques for pain in SCD is lacking.

### Strengths and Limitations

A more objective pain prediction model could significantly help medical providers manage pain crises. As described, data collected from wearable devices can be utilized to improve pain management via advanced machine learning methods. In this analysis, we aimed to build predictive models for pain based on objective, physiologic wearable sensor data. This study is of great value given that the data utilized were obtained from a wearable device and provided minimal to no risk to patients. Furthermore, wearable sensor data were acquired frequently and obtained passively from patients as compared with nurse-documented vitals, which were obtained approximately every 2 hours.

Importantly, wearables and mobile apps (to track symptoms and pain scores over time) paired together to form an mHealth pain prediction system, as in this study, could fairly easily be applied to the inpatient and outpatient settings. mHealth systems are attractive for providers as pain can be tracked on a more frequent basis and can provide more personalized care for patients and potentially prevent ED visits, day hospital visits, and hospital admissions. Further work is needed in this field to continue to develop models with increasing accuracy in predicting pain to help guide management and patient care [47].

There are limitations to our study, including obtaining a convenience sample from our day hospital only and the small number of patients. Patients with SS and SC can be treated the same clinically, but the study included patients with thalassemia who may have a more or less severe phenotype depending on the type of thalassemia. Specific analysis on these patients was not performed for this feasibility study. The study is also limited given that patients might have had underlying medical conditions that could affect HR, and this was not controlled for in our study. In addition, each patient had pain control achieved through individualized pain protocols, which varied among patients and were administered at various intervals. Therefore, it was impossible to control for these pain medications during this initial study. Medications administered, both opioid and nonopioid, may affect vital sign parameters independently (namely, opioids decreasing HR). The administration of pain medication, however, provides an important future opportunity to also evaluate pre- and postadministration objective datasets for pain prediction. Although all patients were in the day hospital either in a chair or bed, their environment was not completely controlled, and HR changes might have occurred with movement in and out of the bed or chair as well as to use the restroom, and these movements were not accounted for. HR can also vary outside of pain when a patient is at rest based on a multitude of different factors including stress, excitement, and breathing.

In addition, our group had to make the assumption that nurse-documented pain scores and patient-reported pain scores in the app were not dissimilar, but this is also an area for further study. One hypothesis would be that the patients could report a lower pain score to the nurse to look tough, but an alternative hypothesis may be a patient elevating their pain score to be given additional medication. There is also the assumption that the physiologic measures from the wearable device are accurate. We attempted to take data averaged over 1 min (with recordings typically every second) to minimize variability. We chose the Microsoft Band 2 because of the ability to acquire the raw data directly from the wearable and because of previous studies showing its relative accuracy. Stahl et al [48] and Shcherbina et al [49] have reported that wrist-based monitors, including the Microsoft Band, provided an accurate measurement of HR in most activity settings. Xie et al [50] further demonstrated that wearable devices had a high accuracy with respect to HR, number of steps, distance, and sleep duration.

Utilizing mobile devices and technology have great promise as we have discussed, but HR data and other physiologic parameters should be interpreted in the clinical context of the patient’s history and exam. For example, a tachycardic patient should be thoroughly evaluated to rule out life-threatening conditions before attributing tachycardia to pain. Although our group has shown that wearable sensor data are feasible to obtain and can be used to create models for predicting pain scores, models and objective vital signs need to be paired with clinical experience and judgment for ideal patient management.

### Conclusions

Future directions include refining the predictive model with a larger dataset. We are continuing to troubleshoot our data extraction procedure to minimize lost data. Furthermore, we could attempt to expand our models by examining patient’s disease severity (related to number of ED visits, day hospital visits, and hospitalizations per year), length of stay in the day hospital, etc, to obtain a more ideal model for pain score prediction. Given that we combined app pain scores with nursing pain scores, further study is needed to determine if these can be treated as similar scores. Related to medication administration, we could examine HR changes before and after medication, time since last dose, total net dose of medications, etc, and attempt to project pain score and the need for medication before the patient requests medication. This would be an essential part of a real-time pain forecasting system and allow a trial that evaluates the timing of administration of additional doses of opioids based on physiologic and objective data alone. Our initial results indicate promise in pursuing each of these efforts, and our study is a valuable addition to ongoing studies investigating how physiologic and objective data can be used to help providers better understand and treat pain.

## Abbreviations

AccZ: acceleration in Z direction
ED: emergency department
GP: Gaussian process
GPR: Gaussian process for regression
GSR: galvanic skin response
HR: heart rate
mHealth: mobile health
NSAID: nonsteroidal anti-inflammatory drug
RMSE: root mean square error
RR: R-R interval
SCD: sickle cell disease
SVM: support vector machines
SVR: support vector machines for regression
TRU-Pain: Technology Resources to Understand Pain
